# Improving Fall Detection Using an On-Wrist Wearable Accelerometer

**DOI:** 10.3390/s18051350

**Published:** 2018-04-26

**Authors:** Samad Barri Khojasteh, José R. Villar, Camelia Chira, Víctor M. González, Enrique de la Cal

**Affiliations:** 1 Sakarya University, 54050 Sakarya, Turkey; samad.khojasteh@ogr.sakarya.edu.tr; 2 Electric, Electronic, Computers and Systems Engineering Department, University of Oviedo, 33003 Oviedo, Spain; vmsuarez@uniovi.es (V.M.G.); delacal@uniovi.es (E.d.l.C.); 3 Computer Science Department, Babes-Bolyai University, 400084 Cluj-Napoca, Romania; cchira@cs.ubbcluj.ro

**Keywords:** fall detection, wearable sensors, elderly people monitoring

## Abstract

Fall detection is a very important challenge that affects both elderly people and the carers. Improvements in fall detection would reduce the aid response time. This research focuses on a method for fall detection with a sensor placed on the wrist. Falls are detected using a published threshold-based solution, although a study on threshold tuning has been carried out. The feature extraction is extended in order to balance the dataset for the minority class. Alternative models have been analyzed to reduce the computational constraints so the solution can be embedded in smart-phones or smart wristbands. Several published datasets have been used in the Materials and Methods section. Although these datasets do not include data from real falls of elderly people, a complete comparison study of fall-related datasets shows statistical differences between the simulated falls and real falls from participants suffering from impairment diseases. Given the obtained results, the rule-based systems represent a promising research line as they perform similarly to neural networks, but with a reduced computational cost. Furthermore, support vector machines performed with a high specificity. However, further research to validate the proposal in real on-line scenarios is needed. Furthermore, a slight improvement should be made to reduce the number of false alarms.

## 1. Introduction

Fall Detection (FD) is a very active research area, with many applications in health care, work safety, etc. [1]. Even though there are plenty of commercial products, the best rated products only reach an 80% success rate [2,3]. There are basically two types of FD systems: context-aware systems and wearable devices [4,5]. FD has been widely studied using context-aware systems, i.e., video systems [6]; nevertheless, the use of wearable devices is crucial because of the high percentage of elderly people and their desire to live autonomously in their own house [7].

Wearable-based solutions may combine different sensors, such as a barometer and inertial sensors [8], 3DACC combined with other devices, like a gyroscope [9], intelligent tiles [10] or a barometer in a necklace [11]. By far, 3DACC is the most used option within the literature [12,13,14,15,16]. Different solutions have been proposed to perform the FD; for instance, a feature extraction stage and SVM have been applied directly in [12,14], using some transformations and thresholds with very simple rules for classifying an event as a fall [15,16,17]. A comparison of classifiers has been presented in [13].

The common characteristic in all these solutions is that the wearable devices are placed on the waist or on the chest. The reason for this location is that it is by far much easier to detect a fall using the sensory system in this placement [18]. Clearly, each location is the best option for some cases, while for other problems, it may not be the best one. For instance, placing the sensor on the waist is valid for patients with severe impairment; however, the requirement to use a belt with some dressing might not be valid in the case of healthy participants. Furthermore, this type of device lacks usability, and people might find it easy to forget them on the bedside table [4,19]. Thus, this research limits itself to use a single sensor, a commercial smart wristband, placed on the wrist.

Furthermore, these previous studies do not focus on the specific dynamics of a falling event: although some of the proposals report good performances, they are just machine learning applied to the FD problem. There are studies concerned with the dynamics in a fall event with sensors located on the waist [20,21,22,23], establishing the taxonomy and the time periods for each sequence. Interestingly, it has been found that the vast majority of the solutions have been obtained using data gathered from simulated falls [3,24]; these studies have found that analyzing the solutions with data gathered from real falls produce a high error rate and rather poor performances. As far as we know, FARSEEING (FAll Repository for the design of Smart and sElf-adaptive Environments prolonging Independent livinG) is the first dataset including data from real elderly persons’ falls [3]. The data have been gathered from patients suffering an impairment illness while using a 3DACC placed either on the thigh or on the lower back. It seems the data must come from the same population in order to record the inherent behavior of the subjects when falling, which may vary with age [25].

Focusing on FD using a wrist-worn bracelet, there are several works published in the literature. Ngu et al. proposed using a BLE link with a smartphone that has access to intelligent web services [26]. Basically, the agent running on the smartphone gathers the 3DACC data stream, analyzing each sliding window with a one step sample. For each sliding window, up to four features are computed and fed to an SVM, which classifies the window as FALLor NOT_FALL. For training the SVM, the authors proposed a training stage for each user, sending the data stream to an intelligent web service to learn the model. Furthermore, the authors proposed a set of ADL to be performed by the user to gather data, including the fall simulation.

In [27], a smartwatch sends the data to a smartphone, where the detection takes place in the smartphone using Kalman filters and the CUMSUMalgorithm [28] using adaptive thresholds. Similarly, a commercial wrist-worn 3DACC wearable was used together with a smartphone to detect falls and inactivity in [29]. In this case, the wearable delivers the processed data to the BLE-linked smartphone, which includes an implementation of WEKA. With the gathered data, an NN model was trained to discriminate between fall and several ADL; also, a threshold-based inactivity detection is continuously updated. As the authors stated, one of the problems is the energy efficiency of the wearable: the data stream, even with BLE, penalizes the battery life. Furthermore, an important problem refers to computing the models in the wearable, which also reduces the autonomy. Moreover, the authors stated the problem of the “heavy dependence on the availability of a smartphone which should therefore always be within a few meters of the user” [29].

A threshold-based solution was analyzed in [30], where up to four different features were computed for each sliding window with one sample shift. Up to 11 thresholds were defined; their values were found experimentally. The authors reported very good results when the alternative ADLs were walking, sitting and other. When the threshold algorithms ran both on the smartphone and on the wrist worn wearable device, the performance was enhanced between 5 and 15%. Although thresholds have been widely used in the literature, having only this type of discrimination might not apply to the general population. Additionally, depending on the availability of the smartphone, this represents a big challenge as the whole FD is computed in this device. A similar solution is proposed in [31], using threshold-based algorithms in both the smartwatch and in the smartphone.

An alternative solution was proposed in [32], where a smartwatch works autonomously to detect falls and send notifications. Threshold-based solutions were proposed assuming that only those falls for which the user faints are the ones to be detected: in the rest of the cases, the user is in charge of calling the ehealth service. Similarly, [27] made use of an Android smartphone to run threshold-based FD. In these latter studies, the authors proposed a continuous analysis of the acceleration magnitude in order to classify the current motion as FALL or NOT_FALL. As the authors stated in these papers, performing more complex models continuously would drain the battery, severely reducing the autonomy of the solution.

In one of the published solutions, Abbate et al. [33] proposed the use of the inherent dynamics of a fall as the basis of the FD algorithm with the sensor placed on the waist. A fall event detection is run continuously based on peak detection; once a peak is detected, a feature extraction is performed, and a feed forward NN classifies the event as FALL or NOT_FALL. A very interesting point of this approach is that the computational constraints for the first two stages are kept moderate so as to be deployed in a wearable device, although this solution includes a high number of thresholds to tune.

The aim of this study is to develop a wrist wearable solution for FD focused on the elderly. A wrist-worn 3DACC on a smart wristband is proposed to enhance the ergonomic skills of the solution. Based on [33], the solution has been implemented and enhanced with (i) an intelligent optimization stage to improve the peak detection, (ii) a dataset balancing stage to avoid biasing the models towards the majority class, (iii) alternative machine learning methods compared to the one originally proposed in order to reduce the computational complexity and promote a longer battery life. Finally, this study makes use of several published datasets, including real falls [3], simulated falls and ADL [34], ADL only [35] and from ADL and simulated epileptic seizures [36]; all of them are using 3DACC, the former with the sensor on the lower back or on the thigh, the three latter with the sensor placed on a wrist. A comparison between real falls suffered by elderly people and falls from young participants in ideal conditions is included to analyze the validity of the results using the simulated falls. Moreover, a complex cross-validation stage, including training, testing and validation, is performed. To our knowledge, this is the first study considering so many different published datasets and a complex scheme of comparison to analyze different FD solutions.

The remainder of this study is organized as follows. Next, the description of the solution proposed in this research is outlined. Section 3 details the experimentation that has been carried out, while Section 4 shows the experiment results and the discussion on them. The study ends with the conclusions drawn from this research.

## 2. Fall Detection with a Wrist-Worn Sensor

**Figure 1:**
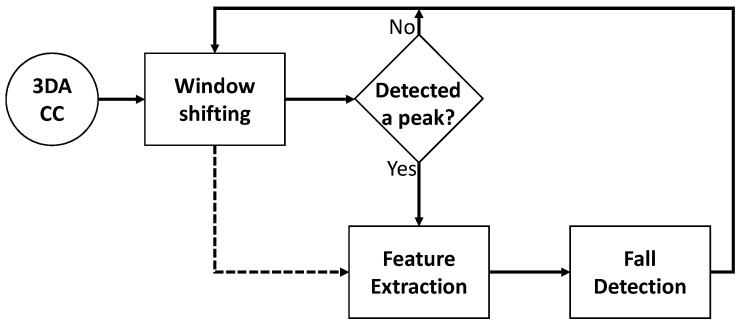
Block diagram of the solution.

The block diagram depicted in Figure 1 is defined in this research, which basically is the proposal in [33]. The data gathered from a 3DACC located on the wrist are processed using a sliding window. A peak detection is performed, and if a peak is found, the data within the sliding window are analyzed to extract several features, which are ultimately classified as FALL or NOT_FALL. The FD block is performed with an AI classifier.

The next subsection describes the method for detecting a peak, as well as the feature extraction, while the method for training the FD block is detailed in Section 2.2. For each case, the proposed modifications are included. A discussion on the most suitable models to be used in this approach is held in Section 2.3. Finally, a new stage is included in the process devoted to the tuning of the peak detection threshold; this stage is explained in Section 2.4.

### 2.1. Feature Extraction Based on the Dynamics of a Fall

**Figure 2:**
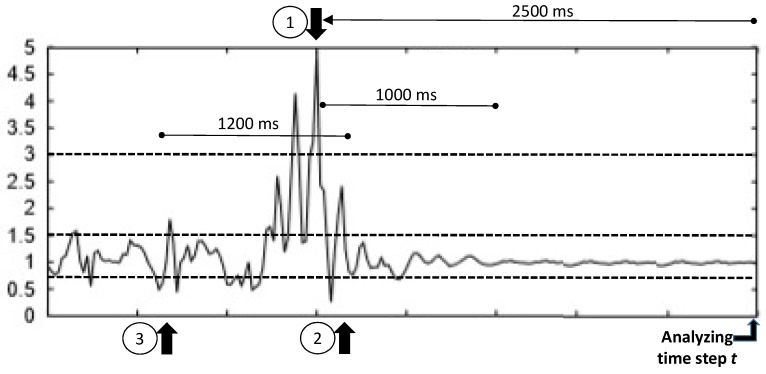
Graph elaborated from [33], showing the evolution of the magnitude of the acceleration in multiples of *g*. Analyzing the signal at time stamp *t*, the peak condition described in the text must be found in order to detect a fall. The *X*-axis represents the time, and each mark corresponds to 500 ms.

Abate et al. [33] proposed the following scheme to represent the dynamics within a fall, so a possible fall event could be detected (refer to Figure 2). Let us assume that gravity is g=9.8 m/s. Given the current times tamp *t*, we find a peak at **peak time** pt=t−2500 ms (Point 1) if at time pt the magnitude of the acceleration *a* is higher than $th_{1}=3\times$ *g* and there is no other peak in the period (t−2500 ms, t) (no other *a* value higher than $th_{1}$). If this condition holds, then it is stated that a peak occurred at pt.

When a peak is detected, the feature extraction is performed, computing for this peak time several parameters and features. The **impact end** (ie) (Point 2) denotes the end of the fall event; it is the last time for which the *a* value is higher than $th_{2}=1.5\times$ *g*. Finally, the **impact start** (is) (Point 3) denotes the starting time of the fall event, computed as the time of the first sequence of an $a<=th_{3}$ ($th_{3}=0.8\times$ *g*) followed by a value of $a>=th_{2}$. The impact start must belong to the interval [ie−1200 ms, pt]. If no impact end is found, then it is fixed to pt+1000 ms. If no impact start is found, it is fixed to pt.

With these three times—is, pt and ie—calculated, the following transformations should be computed:

- Average Absolute Acceleration Magnitude Variation, AAMV=$\sum_{t=is}^{ie}\frac{|a_{t+1}-a_{t}|}{N}$, with *N* the number of samples in the interval.
- Impact Duration Index, IDI=ie−is.
- Maximum Peak Index, $MPI=max_{t\in [is,ie]}\left(a_{t}\right)$.
- Minimum Valley Index, $MVI=min_{t\in [is-500,ie]}\left(a_{t}\right)$.
- Peak Duration Index, PDI=pe−ps, with ps the peak start defined as the time of the last magnitude sample below $th_{PDI}=1.8\times$ *g* occurring before pt and pe the peak end defined as the time of the first magnitude sample below $th_{PDI}=1.8\times$ *g* occurring after pt.
- Activity Ratio Index, ARI, calculated as the ratio between the number of samples not in $\left[th_{ARIlow}0.85\times g,th_{ARIIhigh}=1.3\times g\right]$ and the total number of samples in the 700-ms interval centered in (is+ie)/2.
- Free Fall Index, FFI, the average magnitude in the interval $\left[t_{FFI},pt\right]$. The value of $t_{FFI}$ is the time between the first acceleration magnitude below $th_{FFI}=0.8\times$ *g* occurring up to 200 ms before pt; if not found, it is set to pt−200 ms.
- Step Count Index, SCI, measured as the number of peaks in the interval [pt−2200,pt].

According to the block diagram, each sample of these eight features is classified as a fall event or not using the predefined model. Therefore, this model has to be trained; this topic is covered in the next subsection.

### 2.2. Training the FD Model

Provided there exists a collection of TS with data gathered from real falls or from ADL, a training phase can be proposed to train the FD model. Let us consider a dataset containing $\left\{TS_{i}^{L}\right\}$, with i=1⋯N, *n* the number of TS samples and *L* the assigned label; that is, a sample of this dataset is a $TS_{i}^{L}$ with the data gathered from a participant using a 3DACC on the chosen location, i.e., on a wrist. Let us assume we know a priori whether this $TS_{i}^{L}$ includes or not the signal gathered when a fall occurred; therefore, each TS is labeled as L=FALL or L=NOT_FALL.

Now, let us evaluate the peak detection and the feature extraction blocks for each TS. Whenever a $TS_{i}^{L}$ has no peak, the $TS_{i}^{L}$ is discarded. When a peak is detected for $TS_{i}^{L}$, then the eight features are computed, and label *L* can be assigned to this new sample. Therefore, a new dataset is created with *M* being eight features’ labeled samples, with M≤N. This dataset was used in [33] to train the feed-forward NN.

Nevertheless, it has been found that this solution (i) might generate more than a sample for a single $TS_{i}^{L}$, which is not a problem, and (ii) certainly will generate a very biased dataset, with the majority of the samples belonging to the class FALL. From their study [33], it can be easily seen that the main reason for a 100% detection is this biased dataset.

Consequently, in this research, we propose to include a dataset balancing stage using SMOTE [37], so at least a 40/60 ratio is obtained for the minority class.

### 2.3. Model Complexity and the Battery Life

In [33], Abbate et al. made use of a feed-forward NN. Although the number of hidden neurons was set to seven, using a balanced training dataset as stated in the previous section raises this NN parameter up to 20. Basically, the use of any type of NN is a well-known solution that works quite well in computerized environments [12,14]. Nevertheless, it is known that the higher the number of operations with real numbers the higher the effort a computer has to perform; in the context of wearable and mobile devices, this extra cost matters [38].

In previous research, a comparison between models and their suitability to each possible scenario was presented [36,39]. As it has been shown, those models that include high computation seem to perform better. Actually, K-nearest neighbor outperformed many other solutions; however, its implementation in battery feed devices could drain the battery in a relatively short period of time [40].

Therefore, this research proposes to constrain the models to those that include a low computational impact, reducing complex calculations as much as possible. Actually, in this research, only decision trees and rule-based systems are proposed. These models are based on comparison operations, which are much simpler; the hypothesis is that the obtained results are not going to significantly differ from those of an NN. Finally, to obtain a comparison with state-of-the-art modeling [12,14], we also include the SVM as an alternative.

### 2.4. Tuning the Peak Threshold

As stated in the Introduction of this study, several solutions in the literature are based on thresholds (for instance, [15,20,21,22], among others). In all of these studies, the thresholds were set up based on the data analysis, either by experts or by data engineers through data visualization.

The solution proposed in [33] is not different. Furthermore, several thresholds are used in that study, not only to detect a peak, but also to compute the extracted features. All of them have been fixed by analyzing the gathered data, establishing some typical values for the features for the class FALL.

However, this can be improved by means of computational intelligence and optimization. In this research, we propose to use well-known techniques (genetic algorithms and simulated annealing) to find the most suitable values for these thresholds. This study, in any case, requires not only optimization, but also some design decisions to modify the features. Therefore, for the purpose of this study, we constrain ourselves to focus on the optimization of the peak threshold, which is the most important threshold as it is the one responsible for finding fall event candidates.

## 3. Materials and Methods

### 3.1. Public Datasets

A common way of studying FD is by developing a dataset of simulated falls plus extra sessions of different ADL; all of these TS are labeled and become the test bed for the corresponding study. In this context, a simulated fall is performed by a set of healthy young participants wearing the sensory system, each of them letting him/herself fall towards a mattress from a standing still position.

The vast majority of these datasets were gathered with the sensor attached to the main body, either on the chest, waist, lumbar area or thigh. Interestingly, the UMAFall [34] dataset includes data gathered from 3DACC sensors placed on different parts of the body—ankle, waist, wrist and head—while performing simulated falls; this is the type of data needed in this research as long as the main hypothesis of this study is to perform FD with a sensor worn on a wrist. Furthermore, there is no pattern in the number of repetitions of each activity or fall simulation. Some participants did not simulate any fall; some performed 6 or 9; and one participant simulated 60 falls.

Besides, this research also includes more publicly available datasets. On the one hand, the ADL and simulated epileptic seizure datasets published in [36] are considered because they include a high movement activity, the simulated partial tonic-clonic seizures, followed by a relatively calm period plus some other ADL, all of them measured using 3DACC placed on the dominant wrist. Although this dataset includes neither simulated, nor real falls, it includes activities that share similar dynamics with that proposed for a fall.

Additionally, the DaLiac [35] dataset is also considered in this study. This dataset includes several sensors, one on the wrist and one on the waist, among others. Up to 19 young healthy participants and up to 13 different ADLs are considered, from sitting to cycling.

On the other hand, the FARSEEING dataset [3] is also used for studying the validity of the simulated falls compared with real falls. As stated on the web page, “the FARSEEING real-world fall repository: a large-scale collaborative database to collect and share sensor signals from real-world falls”. Data from 15 participants have been gathered for a total of 22 TS; each TS corresponds to a fall: 7 participants (producing 7 TS) have the 3DACC placed on a thigh, while 8 participants (producing 15 TS) have the 3DACC sensor placed on the lower back. Therefore, this dataset is used to validate the simulated falls, so the extent of the conclusions using the available datasets can be determined. Table 1 summarizes the datasets used in this study.

### 3.2. Dataset Comparison

As mentioned before, the published studies on FD use to base their experimentation on simulated falls with healthy participants, the UMA Fall among them, with an age out of the range of the population on which we focus in this research. In this context, it can be argued that the extrapolation of the conclusions could not be straightforward.

Therefore, a comparison of the signals recorded from the waist from UMA Fall and lower back from FARSEEING is performed, so a conclusion about the similarity of the simulated and the real falls can be drawn. This comparison will consider an exhaustive visual comparison of the signals. To do so, signals of falls from the UMA Fall dataset with the same direction—forward, backward or lateral—will be compared with each of the fall signals coming from a sensor placed on the lower back. The idea is to evaluate whether the dynamics from those TS are similar and if they are similar to that mentioned in [20,33].

### 3.3. A Complete Cross-Validation Scheme

**Figure 3:**
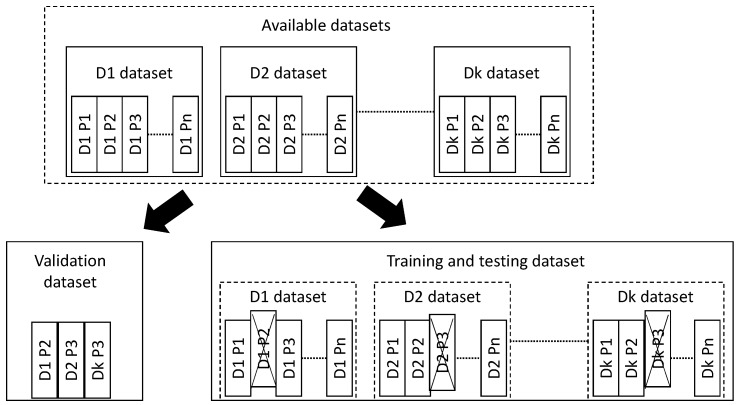
Cross-validation scheme. From the available datasets ($D_{x}$), some participants ($P_{y}$) (and all of their TS) are preserved for validation purposes. The remaining participants and their TS are all conforming to the training and validation dataset.

In this research, a complete cross-validation (cv) scheme is performed, that is including training, testing and validation. Each of these stages includes all the TS from the same individual. In other words, once a participant is chosen to become part of the dataset partition, either validation or training and testing, all of his/her TS are included in that partition (refer to Figure 3). Therefore, none of the TS from a participant included in the validation dataset are used in the training and testing: these two partitions—on one side, the validation, and on the other side, the training and testing—are absolutely unrelated.

The first thing that has been done is choosing the participants from the UMA Fall and the simulated epileptic seizures datasets that are preserved for the validation. Fifteen percent of the participants from each dataset have been chosen to be included in the validation dataset. The remaining participants are assigned to the training and testing dataset.

On this training and testing dataset, cross-validation is performed. Both 10-fold cv and 5 × 2 cv based on participants are performed on a participant’s basis, as well. This means that for each fold, the participants are grouped for training or for testing. Once a participant is grouped for either training or testing, then all of his/her TS are used in the corresponding process. Again, for each fold, the training and testing partitions do not share any participant’s TS; they are completely unrelated.

This scheme is outlined in Figure 3. The advantage of this cv scheme is that it will allow one to evaluate the performance of the solution with unseen participants, those preserved for validation, like would be the case in real life. Furthermore, this scheme allows one to perform training and testing on independent participants. This means that a model is trained with data from a set of participants and then it is tested with data from a different and independent set of participants. Therefore, the training models are tested against data from participants that are totally unseen by them. For sure, this will reduce the performance of the methods, but will allow one to evaluate the robustness of the solutions.

**Figure 4:**
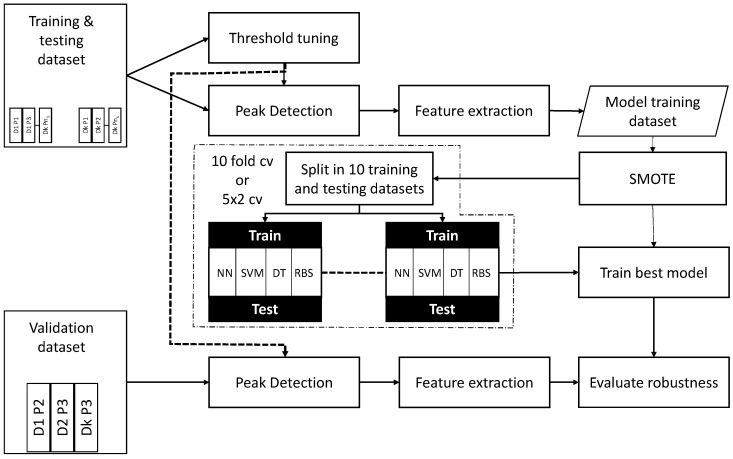
The machine learning process within the cross-validation scheme. The training and testing dataset is used for (i) threshold optimization and (ii) peak detection and feature extraction. The labeled dataset is then used for the machine learning process to find the best modeling option. The best option is then evaluated with the validation dataset once processed so the real performance of the system can be obtained.

The general process is depicted in Figure 4. The training and testing dataset is used for tuning the threshold to perform the peak detection, this optimization process is detailed in Section 3.4. Once the threshold is obtained, then the peak detection takes place. Each TS included in the training and testing dataset is analyzed to find out whether there exists a peak or not. Those detected peaks are analyzed in depth, extracting the eight features and assigning a label: FALL or NOT_FALL.

This new intermediate dataset, called the model training dataset, might be highly imbalanced; therefore SMOTE is applied to obtain a more suitable dataset to use in the learning process. The learning process is detailed in Section 3.5 and includes up to four types of models: feed forward NN, SVM, DT and RBS. The SMOTE configuration will be to obtain a 40–60% representation of the minority class at least. This balanced dataset and the best model configuration found using a grid scheme are used in the training of the model.

Finally, the validation dataset is considered. It goes through the peak detection block, using the optimized threshold, and whenever a peak is found, the feature extraction stage is executed. Finally, the eight features are classified using the best model found in the previous stage. A TS from the validation dataset will be classified as FALL if a peak is detected and the subsequent classifier outputs the FALL label; otherwise, the TS will be assigned the label NO_FALL.

To evaluate this validation stage, and every classification result in this study, the standard measurements accuracy, Kappa factor, precision, sensitivity, specificity and the geometric mean of these two latter will be computed. In order to compute the TP, TN, FP and FN, each TS is labeled with FALL if it includes a fall event; otherwise, it is labeled as NOT_FALL. Each TS is evaluated using each of the classifiers; a label FALL is assigned to the TS whenever a peak is detected and the corresponding output of the classifier is FALL; otherwise, the TS is labeled as NOT_FALL. Then, the following formulas hold.

(1) $$Acc=\frac{TP+TN}{TP+TN+FP+FN}$$

(2) $$P_{0}=TP+TN$$

(3) $$P_{e}=\left(TP+FN\right)\times \left(TP+FP\right)+\left(TN+FP\right)\times \left(TN+FN\right)$$

(4) $$K=\frac{P_{0}-P_{e}}{1-P_{e}}$$

(5) $$Se=\frac{TP}{TP+FN}$$

(6) $$Sp=\frac{TN}{TN+FP}$$

(7) $$Pr=\frac{TP}{TP+FP}$$

(8) $$G=\sqrt{\frac{TP}{TP+FN}\times \frac{TN}{TN+FP}}$$

### 3.4. Tuning the Peak Detection Threshold

A peak is detected whenever the acceleration magnitude is higher than 3× *g* as defined in [33] when the sensor is located on the waist. However, is this a valid value when the sensor is located on a wrist? This question will be answered using two metaheuristics: Genetic Algorithms (GA) and Simulated Annealing (SA).

The peak threshold is encoded as a real value ranging from 2.0 to 3.5. As explained in the dataset comparison experiments, these values were collected from the analysis of the TS from the UMA Fall gathered with the sensor on the wrist; for the sake of brevity, these TS are not plotted. The encoded real value represents a possible solution for both GA and SA approaches. The quality of the solution is evaluated using a fitness function based on the sensitivity and specificity obtained by the classification measurements generated using the current peak threshold. The fitness function used to guide the search process of the metaheuristics is the geometric mean of the specificity and the sensitivity, that is f(x)=G(x); see Equation (8).

The GA starts with a population of randomly-generated individuals. Each generation, convex crossover is applied with a certain probability between each individual and a mate selected using a binary tournament. The resulting offspring replaces the first parent if it has a better fitness value. Gaussian mutation is then applied to the current individual with a fixed probability. Mutation perturbs the peak threshold using a zero-mean Gaussian distribution, and the new obtained value is allowed to replace the current individual. This unconditioned replacement enhances the diversity of the population and benefits the search process. The parameter setting is performed with the aim to keep the number of fitness evaluations as low as possible in order to avoid high computational cost. To this end, the peak threshold optimization using GA is based on a population size and generation number of 10, crossover probability of 0.8 and mutation probability of 0.2.

The SA algorithm is based on a single solution initialized with a random value in the considered range [2.0,3.5]. The neighborhood of a solution is defined based on the Gaussian mutation. A new solution *y* selected from the neighborhood of a current solution *x* is accepted as the new current solution if it has better fitness or with a probability defined according to the SA approach (as given below).

(9) $$P^{accept}=e^{\frac{f(y)-f(x)}{T}}$$

The probability of accepting a new solution from the neighborhood that does not improve the current fitness value depends on the difference between the fitnesses of the two solutions and on an SA parameter called temperature (denoted by *T* above). The cooling scheme for the temperature is based on a simple iterative function that returns the current *T* multiplied by a constant value α. For each value of *T* starting from the initial temperature to the minimum temperature, several iterations are allowed to select a new neighboring solution. In the current parameter setting, the value of *T* starts at 1.0, the minimum temperature is 0.1, the value of α is 0.9 and the number of iterations is set to 5. Parameter values for both GA and SA have been selected based on some preliminary experiments according to the results obtained and the computational cost.

### 3.5. Model Learning

The original solution proposed in [33] made use of a feed-forward NN with 7 hidden neurons. However, in that original paper, the authors did not balance the model training dataset. In our experience, the feature extraction domain was clearly unbalanced toward the FALL label, so obtaining good results for the FALL label does not guarantee a good performance as the specificity might be really poor. Further, if this approach were to be deployed on a smart wristband or similar device, it would be advisable to use low computational models.

Therefore, in this study, several different models are proposed: the feed-forward NN, support vector machines (SVM), C5.0 decision trees (DT) and C5.0 rule-based systems (RBS). The former is the one proposed in the original work, and the two latter are simpler models based on C4.5. Alternatively, SVM is proposed as an alternative state-of-the-art modeling technique that has been applied in FD [12,14]. All of them are implementations included in the caret package for R [41,42].

For each model technique, a grid search for the most interesting parameters will be performed after the balancing stage, even for the NN as long as the model training dataset has changed from that originally published.

## 4. Results and Discussion

### 4.1. Dataset Comparison

**Figure 5:**
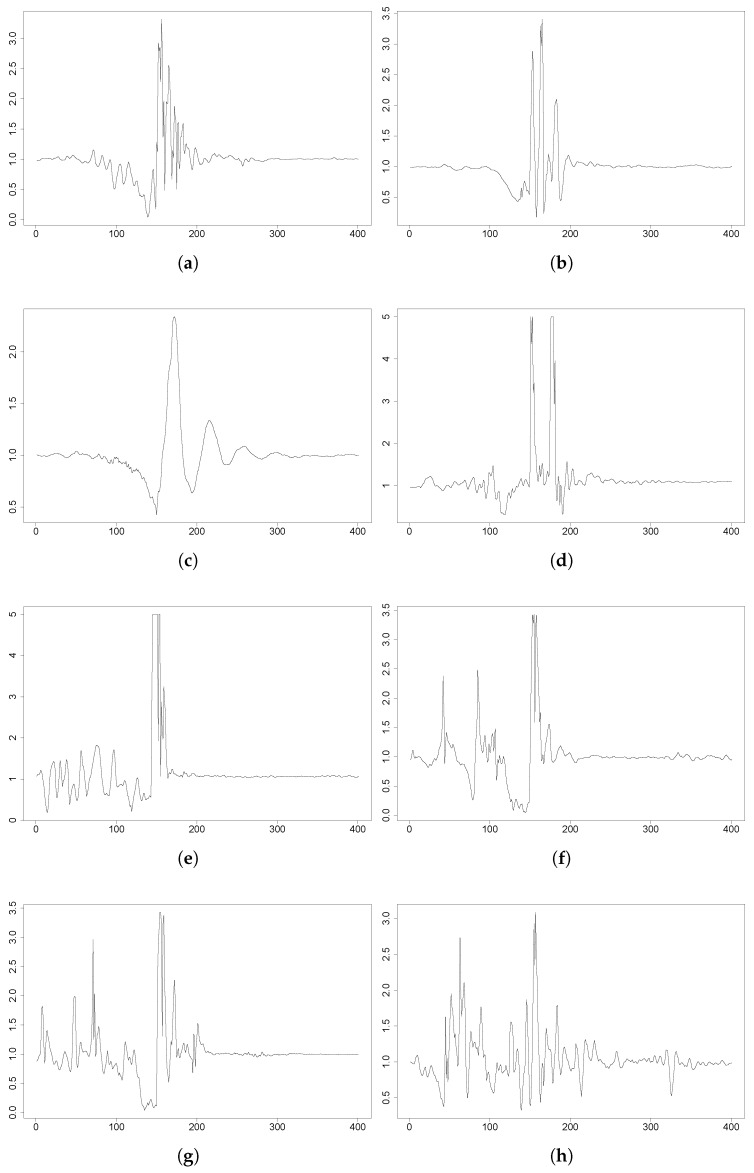
FARSEEING plots of some falls depicting the magnitude of the acceleration during a fall; 400 samples in 4 s. From left to right and top to bottom: F1, F2, F3, F4, F5, F6, F7 and F8. F3 shows a peak below 3× *g*. In all the cases, the sensor is located on the lower back. (**a**) F1: backward fall, 3DACC ∈[0.0,3.5]× *g*. (**b**) F2: forward fall, 3DACC ∈[0.0,3.5]× *g*. (**c**) F3: forward fall, 3DACC ∈[0.0,3.0]× *g*. (**d**) F4: forward fall, 3DACC ∈[0.0,5.2]× *g*. (**e**) F5: forward fall, 3DACC ∈[0.0,5.2]× *g*. (**f**) F6: backward fall, 3DACC ∈[0.0,3.6]× *g*. (**g**) F7: backward fall, 3DACC ∈[0.0,3.6]× *g*. (**h**) F8: unknown direction, 3DACC ∈[0.0,3.3]× *g*.

The FARSEEEING dataset includes up to 15 falls from elderly people using a 3DACC placed on the lower back; for each of them, there might be a break in the circumstances of the fall event. This context information is included in Table 2 with the corresponding ID within this research and within the FARSEEING dataset. Furthermore, in Figure 5, the evolution of the acceleration magnitude is plotted for F1to F8. Although for the majority of the subjects, the 3× *g* threshold remains valid, some subjects perform with lower peak values; i.e, F3 in the figure. Furthermore, F9 has a peak value below the Abbate et al. threshold, though it has not been included for the sake of space.

**Figure 6:**
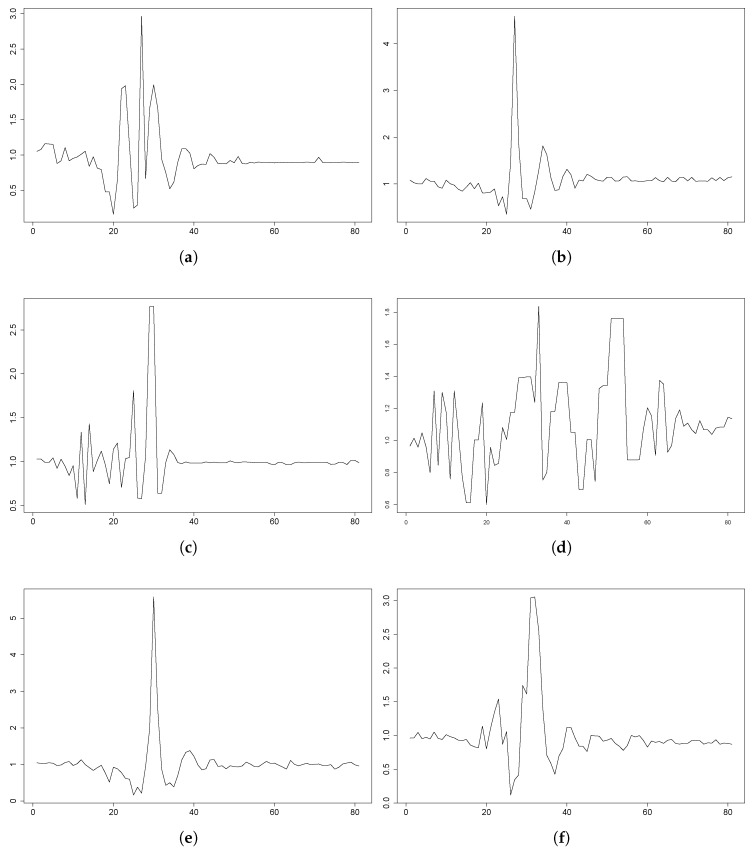

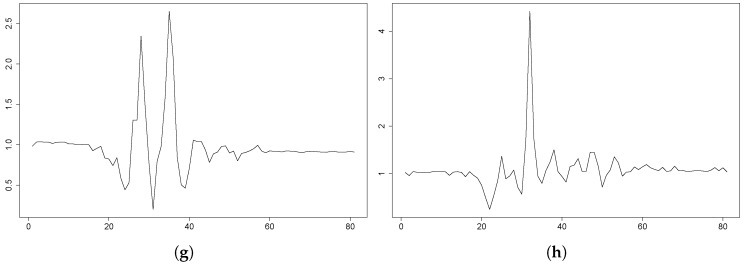
UMA Fall plots for some falls that behave differently from what was expected. The plots depict the magnitude of the acceleration during a fall in a period of 4 s (80 samples). The data come from the 3DACC sensor on the waist. (**a**) P1: forward fall, 3DACC ∈[0.0,3.2]× *g*. (**b**) P1: backward fall, 3DACC ∈[0.0,5.0]× *g*. (**c**) P12: lateral fall, 3DACC ∈[0.0,2.8]× *g*. (**d**) P12: backward fall, 3DACC ∈[0.0,2.0]× *g*. (**e**) P9: lateral fall, 3DACC ∈[0.0,6.0]× *g*. (**f**) P9: forward fall, 3DACC ∈[0.0,3.2]× *g*. (**g**) P15: forward fall, 3DACC ∈[0.0,2.8]× *g*. (**h**) P15: backward fall, 3DACC ∈[0.0,4.5]× *g*.

Besides, Figures and depict several fall events from the participants in the UMA Fall dataset. In these figures, $P_{x}$ refers to the corresponding participant in that dataset, and the plots include the 3DACC magnitude (see Equation (10)) data from the sensor on the waist. Most of the participants did fairly similar to the hypothesis of dynamics and also the thresholds in [33]. Nevertheless, there were also several exceptions; see Figure 6. For instance, Participants 1, 2 and 15 seem to have been falling with fear: their movements were clearly slower. For these participants, some tests were fair, even with a remarkable magnitude value higher than the 3× *g* threshold; for some other tests, they performed gently. In some tests, the participant behaved really differently, with the evolution of the magnitude of the acceleration having a totally different shape: Participant 12, the backward fall included in the figure.

(10) $$\hat{a}=\sqrt[{2}]{a_{x}^{2}+a_{y}^{2}+a_{z}^{2}}$$

**Figure 7:**
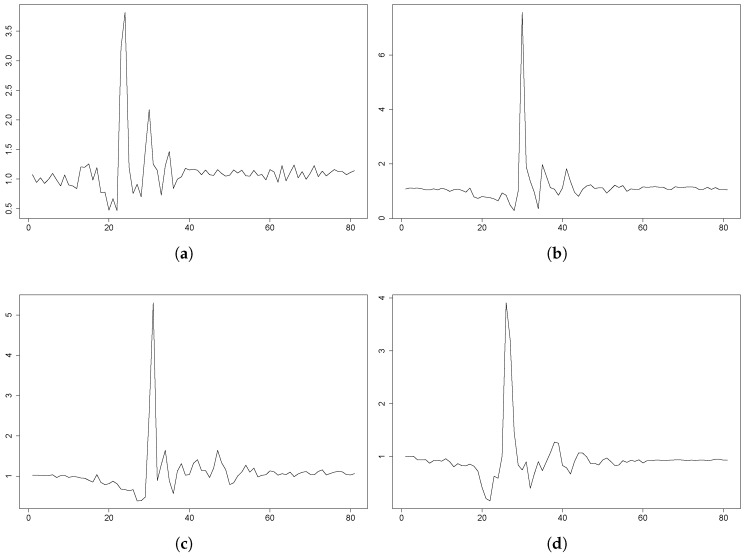

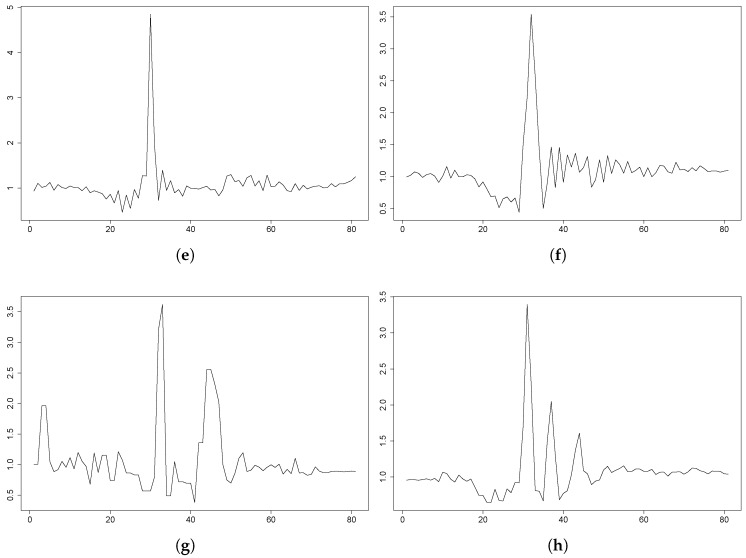
UMA Fall plots for some fall events from participants behaving as expected. The plots depict the magnitude of the acceleration during a fall in a period of 4 s (80 samples). The data come from the 3DACC sensor on the waist. (**a**) P1: backward fall, 3DACC ∈[0.0,4.0]× *g*. (**b**) P1: backward fall, 3DACC ∈[0.0,8.0]× *g*. (**c**) P2: lateral fall, 3DACC ∈[0.0,5.5]× *g*. (**d**) P2: forward fall, 3DACC ∈[0.0,4.1]× *g*. (**e**) P5: backward fall, 3DACC ∈[0.0,5.0]× *g*. (**f**) P5: backward fall, 3DACC ∈[0.0,3.5]× *g*. (**g**) P12: forward fall, 3DACC ∈[0.0,3.8]× *g*. (**h**) P15: lateral fall, 3DACC ∈[0.0,3.5]× *g*.

However, the majority of the simulations behaved as expected (refer to Figure 7). As seen in these plots, with the independence of the fluctuation of the signal due to the different sampling frequencies, the dynamics can be considered similar to those shown in FARSEEEING, accomplished to some degree with the dynamics proposed in [33]. Still, some differences in this issue can be observed.

On the one hand, the peak threshold is valid for the majority of the cases, but some of the TS behaved under that limit. This will produce a false negative, that is there will be undetected falls. This is the reason why in this research an optimization stage is included in order to tune the peak threshold. The range of possible candidates is defined with the smallest peak threshold found for all the TS from the UMA Fall dataset for the sensor on the wrist: this value has been found to be 2.5× *g*; therefore, the lower limit was set to 2.0× *g*. The upper limit of the range is defined as a relatively large threshold, which was estimated as 3.5× *g*.

On the other hand, the FARSEEING includes some TS that cover walking and a sudden fall; the TS obtained for these cases may change the time periods mentioned in [33]. Moreover, each subject and participant has a different reaction speed. These two ideas must be reconsidered in future work to revisit the definition of the extracted features.

Due to the fact that there were visual differences in the behavior of the different datasets, and also because it would allow a better comprehension of the similarities between the simulated and real falls, a comparison between the TS from the FARSEEING and from the UMA Fall datasets is performed using the algorithm and thresholds proposed in [33]. Table 3 shows the mean and the standard deviation of the values of the extracted features for the TS that include a fall event. Using the Shapiro normality test, it was found that not all features follow a normal distribution; thus, a Mann–Whitney–Wilcoxon test was used to evaluate whether the features from each dataset belonged to the same distribution or not. These results are included in Table 3, as well.

As can be seen, the results clearly show the differences between the simulated and the real falls. This is a very relevant finding as it is normal in the literature to use simulated falls in the evaluation of FD algorithms: now, it is found that there is evidence to not accept simulated falls as valid. Although these differences might be explained because the participants in the FARSEEING datasets suffer from impairment illnesses, it is clear from the obtained results that what is found out in the next subsections needs to be validated in real scenarios, with participants from the population in focus living independently, but keeping a log of any possible fall that might happen so real data could be gathered.

Notwithstanding the differences between the simulated and the real fall datasets found so far, we have no other option than to use the simulated fall dataset because, to the best of our knowledge, there are no publicly available real fall datasets using a 3DACC sensor placed on a wrist. Nevertheless, further research will be needed, as explained before.

Moreover, there are some issues in the UMA Fall dataset that need further addressing. When people fall, they use their arms to protect themselves and to try to grab something to avoid falling. Therefore, there will be much more movement variability, from those who fall without moving the arms to those that frantically try not to fall. Research with sensors worn on the wrist and in real scenarios will be needed.

### 4.2. Threshold Optimization

The GA and SA algorithms have been run 10 times based on the parameter setting given in Section 3.4. The results obtained have been analyzed according to the fitness function defined to guide the search process. The dataset used in this threshold optimization, following the experiment scheme shown in Figure 4, was the training and testing dataset.

The best fitness value generated by the GA is 0.870 for the peak threshold values 3.09629, 3.09632 and 3.0971. The average fitness over 10 runs is 0.8695, which only slightly deviates from the best run. The best thresholds detected by GA are mostly in the fitness range from 3.093 to 3.109 with a median value of 3.09590.

The SA algorithm obtains similar results to the GA. The best fitness value is 0.869 obtained for the peak threshold values 3.0936, 3.0921, 3.0940 and 3.0984. The average fitness for the 10 SA runs considered is 0.868, which is, as in the case of GA, near the best value obtained. This indicates a stable performance for both algorithms over the independent runs. Most peak values detected by SA range from 3.078 to 3.093 with a median value of 3.09290. As already emphasized, these are fitness values obtained based on the training and testing data. As can be noticed, GA and SA trigger similar results both in terms of the best and average fitness values, as well as the median peak threshold values.

After these optimization stages, and also by the visualization stage performed in the previous subsection, the following thresholds will be compared:

- th25=2.5× *g*: as the minimum value to detect any peak in the datasets.
- th3=3.0× *g*: the original proposal from [33].
- th309=3.09290× *g*: the median of the values obtained from the SA optimization. The median value obtained from the GA runs was 3.09590× *g*, which is quite a similar value; for the sake of the length, only the SA optimized value will be analyzed.

### 4.3. Model Training and Cross-Validation Results

Recall that the experimentation design included several published datasets; these datasets were split into training, testing and validation. When splitting, the participants (and all of the TS gathered from them) were assigned either to the training and testing or to the validation datasets. Furthermore, the majority of the available datasets gathered using a wrist-worn 3DACC do not include fall events but ADL, including jumping, simulated seizures or running, among others; this results in a more balanced feature extraction dataset than if only a single dataset were used. Nevertheless, a SMOTE stage was performed to guarantee 40% minority samples in the training and testing dataset.

The best parameter subset was obtained for each pair of threshold and model type using a grid search. The obtained parameter subsets are shown in Table 4 for the feed forward NN, in Table 5 for SVM and in Table 6 for both the decision tree and the rule-based system based on C5.0.

**Figure 8:**
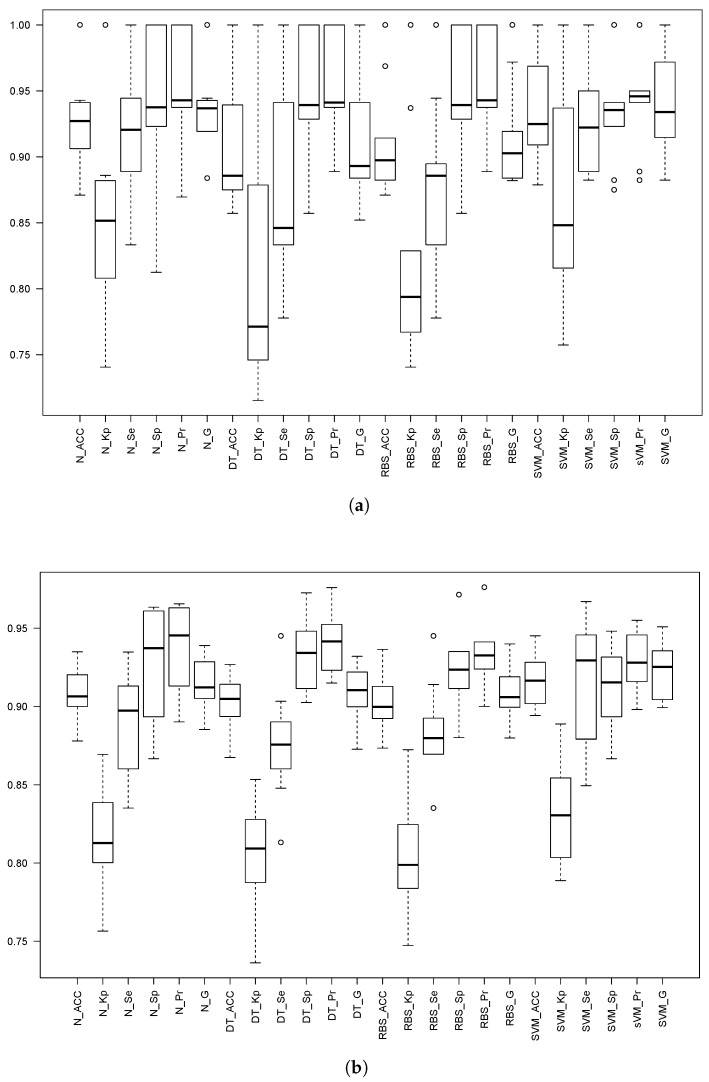
Box plots of the different statistics for the three models when the threshold is set to 2.5× *g*. The prefixes N_, SVM_, DT_ and RBS_ stand for the NN, SVM, Decision Trees C5.0 and Rule-Based System C5.0 models. The statistics are: Accuracy (Acc), Kappa factor (Kp), Sensitivity (Se), Specificity (Sp), Precision (Pr) and the Geometric mean (G), all of them computed using Equations (1) to (8). (**a**) Ten-fold cv; (**b**) 5 × 2 cv.

**Figure 9:**
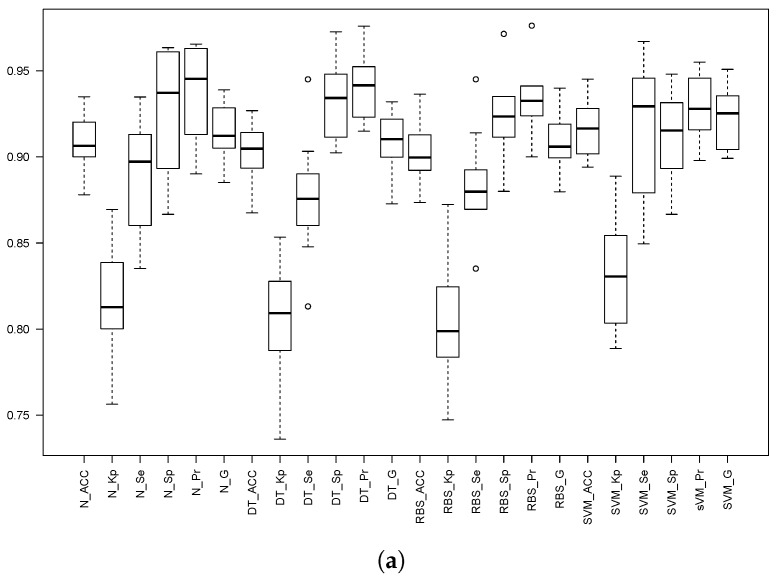

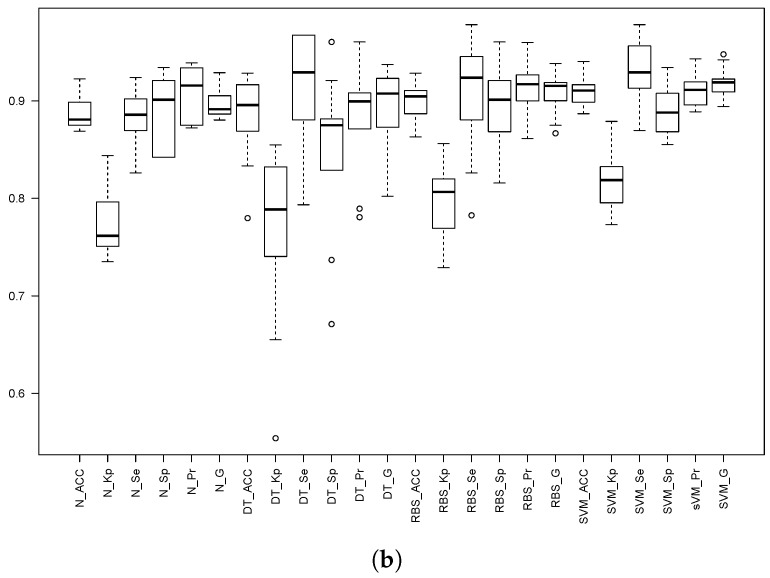
Box plots of the different statistics for the three models when the threshold is set to 3.0× *g* (upper part) and 3.09290× *g* (lower part) with the 5 × 2 cv. The prefixes N_, SVM_, DT_ and RBS_ stand for the NN, SVM, Decision Trees C5.0 and Rule-Based System C5.0 models. The statistics are: Accuracy (Acc), Kappa factor (Kp), Sensitivity (Se), Specificity (Sp), Precision (Pr) and the geometric mean (G), all of them computed using Equations (1) to (8). (**a**) th=3.0× *g*. (**b**) th=3.09290× *g*.

Both 10-fold cv and 5 × 2 cv were performed, and the obtained results are depicted and shown in Figure 8 and Table 7 and Table 8 for threshold *th*25. For both thresholds *th*3 and *th*309, only the 5 × 2 cv results are included for the sake of both readability and space; Table 9 shows the 5 × 2 cv for threshold *th*3, and Table 10 shows the 5 × 2 cv results for threshold *th*309. Finally, Figure 9 depicts the boxplots for 5 × 2 cv for both *th*3 and *th*309.

Recall that these results regard the feature extraction dataset obtained for the corresponding threshold. This means that, in this stage of the experiment, we are only considering that if a peak is found we could correctly label it to belong to the FALL or NOT_FALL class. Thus, this would allow us to choose the most suitable model, if enough evidence is found.

In general, the results for 10-fold cv are better due to the differences in the number of samples contained in the training and testing datasets; however, the same behavior of the statistics can be observed. This is the reason why only the results for 5 × 2 cv are shown in the remainder of this subsection.

We have statistically compared the different methods for each of the thresholds. To do so, we have used the analysis of variance and Tukey honest significance differences, both tools included in R. With a confidence level of 95%, it has been stated that:

- For *th*25, SVM outperforms in sensitivity the other methods. However, in the remainder of the classifier performance measurements, all the methods are comparable.
- For *th*3, all of the methods are comparable except when using the sensitivity. With this measurement, NN is outperformed by SVM, DT and RBS.
- For *th*309, all the methods are comparable for all the measurements.

As a conclusion of this stage, we can state that:

- The different models are totally comparable, and there is no evidence that one of the combinations outperforms the others.
- In this scenario of similar behavior, the DT or the RBS might be advisable due to their simplicity and tuning capability. However, SVM are at least as interesting as these two models.
- No threshold has been observed better than the others. The original threshold proposed in [33] is just in the middle compared with the performances obtained for *th*25 and *th*309.
- The decrease in the performance from the 10-fold cv to the 5 × 2 cv suggests that the validation results might be significantly worse, as the participants chosen for validation have not been presented to the models, representing the performance in real scenarios in the case of deploying the solution.

Henceforward, there is no clear winner from this comparison, neither with the threshold values, nor with the models. Thus, the next stage of the experimentation, which will evaluate the overall performance of the pair <threshold, model>, will be the definitive phase in this research.

### 4.4. Final Validation

In this stage, the performance of the whole solution will be evaluated. To do so, for each threshold, a model will be learned using the corresponding best parameter subset and the full training and testing datasets. With these models, the following algorithm is performed:

For each participant included in validation
 For each TS from the current participant
   If a peak is found using the currently chosen threshold
     Extract the features
     Predict the class using the corresponding model
     Update the classifying statistics according to the TS label
   Otherwise
     Update the classifying statistics according to the TS label

The obtained results are included in Table 11 and Table 12. The former shows the confusion matrices for each combination of threshold and model. The latter shows the classifying performance of the whole solution.

In our opinion, the confusion matrices obtained for the *th*25 threshold, independently of the model, show a performance where (i) the number of false alarms is higher for the NN and RBS and (ii) there are several undetected fall events. The relevance of an undetected fall event makes the *th*25 threshold the worst candidate.

Increasing the threshold has a clear impact on the number of undetected fall events. However, the false alarm number varies from one case to the other: the number of false alarms and the corresponding specificities suggest that further research is needed to tackle this problem. More importantly, for the *th*309, two of the models did detect all the fall events, which suggests this *th*309 threshold learned from the optimization stage may be considered the best solution.

Furthermore, the comparison of the two main models (NN and RBS) shows this latter as more robust and reliable as the number of false alarms is about one third of that reported for the NN.

Besides, the SVM performance is really good if we consider the small number of false alarms (showing a very good specificity indeed), although it was not able to detect all the falls.

However, there is no real evidence about how this solution would perform with elderly people because (i) the intensity level of the ADL is expected to be smaller for this population, which favors the solution, indeed, (ii) there are no real fall datasets with the sensor placed on one wrist, which go against this solution, and (iii) adapting the thresholds for each individual is not addressable in the current design. Furthermore, there would be differences between the evolution of the 3DACC TS for healthy elderly people and those obtained for, say, elderly people with impairments. These reflections lead us to conclude that a solution should be independent of the user intensity level and easier to tune and adapt to the current user. Moreover, gathering data from the elderly population would help in obtaining a more representative dataset. In those cases, like in faints, where there is not enough data, mimicking the faints with human-like flexible mannequins can also help.

## 5. Conclusions

This research focuses on fall detection for elderly people. Several solutions were studied, one of them was chosen for deployment and improvement with the premise of a reduced computational cost because it has to be implemented on wearable sensors. A threshold-based peak detection plus an NN stage to label the features extracted from the data has been extended with (i) an optimization stage to find the best threshold candidate, (ii) an SMOTE stage to balance the classes in the feature extraction domain and (iii) alternative classifiers with reduced computation and higher adaptability.

The experimentation includes several published datasets: the FARSEEING dataset that includes real falls gathered from a 3DACC placed on the lower back of patients suffering from some impairment illnesses, the UMA FALL including simulated falls and ADL with several sensors and locations, the DaLiac including ADL and the simulated epilepsy, including ADL and simulated seizures; these two latter datasets gathered the data from a 3DACC placed on a wrist. After a comparison of the falls included in FARSEEING and in UMA Fall, it has been found that simulating falls might not represent the real movements. Therefore, using simulated data might help in evaluating a solution, but extra research with data from real falls will be needed in order to validate a solution.

The threshold optimization introduced did not show a clear advantage with regard to neither the original proposed in [33], nor the manually chosen one. However, SVM, RBS and DT were found comparable for almost all the cases. Besides, SVM was the modeling technique that performed with better specificity, producing the smallest amount of false alarms.

More research is needed to find a solution that performs independently of the intensity level of the user. Furthermore, the relevance of the wrist orientation in the FD must be evaluated. Moreover, a dataset gathered from elderly people using the sensor on a wrist and including real falls is needed. Additionally, using mannequins would enrich the fall detection dataset. Finally, the use of different oracles for different types of falls, like faints, for instance, might be needed to cope with all the possible sources of fall events to detect. Perhaps introducing ensembles can enhance the final results, but always keeping in mind the battery life of the wearable smartwatches.

## Figures and Tables

**Table 1:** Descriptions of the different datasets used in this research. Columns NP, NF and NR stand for the Number of Participants, the Number of Falls in the dataset and the Number of goes for each ADL, respectively. A question mark (?) means that it is not a regular value. The sampling frequency used in gathering the dataset is stated in Hz in the frequency column (Fqcy).

Dataset	NP	NR	NF	Fqcy	Description
UMAFall [34]	17	?	208	20	Includes forward, backward and lateral falls, running, hopping, walking and sitting. Neither do all the participants have every type of activity, nor the same number of goes. Sensors on the wrist, waist, ankle, chest and trouser pocket.
DaLiac [35]	19	1	0	204.8	Sitting, standing, lying, vacuuming, washing dishes, sweeping, walking, up and down stairs, using a treadmill, cycling and rope jumping. Sensors on the wrist, waist, ankle and chest.
Epilepsy [36]	6	10	0	16	Walking at different paces, running, sawing and simulating epileptic partial tonic-clonic seizures. Sensor on the dominant wrist.
FARSEEING [3]	15	?	22	100	22 real falls recorded from 15 elderly people; each of the files might include a description: context of the fall event, where the sensor was located, etc. Sensor located either on the lower back or on the thigh.

**Table 2:** FARSEEING dataset: Context information of each fall event for subjects wearing a 3DACC placed on the lower back. ID and FRSIDare the Identification within this study and that given in the dataset, respectively. The context is extracted from the FARSEEING documentation [3].

ID	FRSID	Context
F1	17744725-01	Female. After walking with a wheeled walker, stood behind a chair, then fell
		backwards on the floor. Standing, backward fall.
F2	38243026-05	Female. Fell forward while bending down to fix a shoelace. Bending down,
		forward fall.
F3	42990421-01	Male. Wanted to pick up an object from the ground. Bending down, forward fall.
F4	42990421-02	Male. Got up from the chair and wanted to walk. Transferring from
		sitting to standing, forward fall.
F5	42990421-03a	Male. When trying to move to the side, the wheeled walker fell forward.
		Freezing of movement. Walking, side-forward fall.
F6	72858619-01	Female. Went to the table in the dining room. Fell down backwards on
		the buttocks. Standing, backward fall.
F7	72858619-02	Female. Person held on the wall, then fell down backwards on the buttocks.
		Standing, backward fall.
F8	72858619-02	Male. Fell during walking. Walking, unknown fall direction.
F9	74827807-07	Male. Walking, then fell down in front of the entrance of the house.
		Walking, unknown fall direction.
F10	79761947-03	Female. Changing the hip protector, fell backwards on the ground and
		hit the toilet. Standing, backward fall.
F11	91943076-01	Female. Changing the hip protector, fell backwards on the ground and
		Walking, unknown fall direction.
F12	91943076-02	Female. Walking, then fell down opening the door in the entrance hall.
		Walking, unknown fall direction.
F13	96201346-01	Female. Walking to the bathroom, stopped with freezing and fell from
		standing position. Walking, left-backward fall.
F14	96201346-02	Female. Standing at the wardrobe, wanted to walk backwards and then fell
		on buttocks. Standing, backward fall.
F15	96201346-05	Female. Walking backwards from the wash basin, then fell backwards.
		Standing, backward fall.

**Table 3:** Comparison between FARSEEING (sensor on the lower back) and UMA Fall (sensor on the waist) datasets: the mean and standard deviation (Std) of the features computed for all the TS that correspond to a fall. The last row shows the *p*-values from the Mann-Whitney-Wilcoxon test (MWW test), showing that the features from FARSEEING and UMA Fall do not follow the same probability distribution.

**Mean**	**AAMV**	**IDI**	**MPI**	**MVI**	**PDI**	**ARI**	**FFI**	**SCI**
FARSEEING	0.1592	1.2225	4.2345	0.6363	1.2160	0.5933	1.5660	4.7170
UMA Mob	0.5155	1.4619	5.9534	0.4943	1.5911	0.3475	1.3607	2.3730
**Std**	**AAMV**	**IDI**	**MPI**	**MVI**	**PDI**	**ARI**	**FFI**	**SCI**
FARSEEING	0.1701	2.5817	2.6419	0.3002	2.5817	0.3376	0.8590	3.8098
UMA Mob	0.3889	2.002	2.928	0.2358	1.4173	0.3142	0.8768	1.5238
**MWW test**	**AAMV**	**IDI**	**MPI**	**MVI**	**PDI**	**ARI**	**FFI**	**SCI**
*p*-value	2.20 × 10${}^{-}$${}^{1}$${}^{6}$	1.68 × 10${}^{-}$${}^{6}$	1.54 × 10${}^{-}$${}^{7}$	0.0009	1.04 × 10${}^{-}$${}^{1}$${}^{0}$	4.53 × 10${}^{-}$${}^{6}$	0.0022	4.70 × 10${}^{-}$${}^{5}$

**Table 4:** Best parameter set found for the feed forward NN and for each threshold.

Threshold	Size	Decay	Max. Iter.	Abs. Tol.	Rel. Tol.
2.5	20	1 × 10${}^{-}$${}^{6}$	500	4 × 10${}^{-}$${}^{6}$	1 × 10${}^{-}$${}^{1}$${}^{0}$
3.0	20	1 × 10${}^{-}$${}^{4}$	1000	4 × 10${}^{-}$${}^{6}$	1 × 10${}^{-}$${}^{9}$
3.09290	20	1 × 10${}^{-}$${}^{3}$	1000	4 × 10${}^{-}$${}^{6}$	1 × 10${}^{-}$${}^{9}$

**Table 5:** Best parameter set found for the SVM and for each threshold.

Threshold	Sigma	C
2.5	0.2	4.5
3.0	0.2	0.7
3.09290	0.20	1.0

**Table 6:** Best parameter set found for the Decision Tree (DT) and Rule-Based System (RBS) based on C5.0, for each threshold.

Threshold	Model	Winnow	Trials	CF	Bands	Fuzzy Threshold
2.5	DT	FALSE	15	0.5	0	FALSE
2.5	RBS	FALSE	20	0.5	0	FALSE
3.0	DT	FALSE	20	0.05	0	FALSE
3.0	RBS	FALSE	15	0.20	0	FALSE
3.09290	DT	TRUE	10	0.5	0	FALSE
3.09290	RBS	TRUE	5	0.05	0	TRUE

**Table 7:** Results obtained from the 10-fold cv when the threshold is set to 2.5× *g*: the different statistics are the Accuracy (Acc), Kappa factor (Kp), Sensitivity (Se), Specificity (Sp), Precision (Pr) and the Geometric mean (G), all of them computed using Equations (1) to (8). The models are feed forward NN, Support Vector Machine (SVM), decision trees learned with C5.0 (DT) and Rule-Bases systems learned with C5.0 (RBS).

	**NN**						**DT**					
**Fold**	**Acc**	**Kp**	**Se**	**Sp**	**Pr**	**G**	**Acc**	**Kp**	**Se**	**Sp**	**Pr**	**G**
1	0.9091	0.81564	0.92857	0.89474	0.86667	0.89709	0.78788	0.5777	0.85714	0.7368	0.70588	0.77784
2	0.8611	0.71154	0.86364	0.85714	0.90476	0.88396	0.88889	0.7534	1.00000	0.7143	0.84615	0.91987
3	0.9211	0.84034	0.83333	1.00000	1.00000	0.91287	0.94737	0.8939	0.88889	1.0000	1.00000	0.94281
4	0.9032	0.80503	1.00000	0.80000	0.84211	0.91766	0.87097	0.7406	0.93750	0.8000	0.83333	0.88388
5	0.9375	0.85520	0.91304	1.00000	1.00000	0.95553	0.81250	0.5915	0.78261	0.8889	0.94737	0.86106
6	0.9118	0.82353	0.88235	0.94118	0.93750	0.90951	0.94118	0.8824	0.94118	0.9412	0.94118	0.94118
7	0.8387	0.68041	0.75000	1.00000	1.00000	0.86603	0.80645	0.6092	0.75000	0.9091	0.93750	0.83853
8	0.8438	0.68750	0.78947	0.92308	0.93750	0.86031	0.87500	0.7470	0.84211	0.9231	0.94118	0.89026
9	0.9714	0.93783	0.92308	1.00000	1.00000	0.96077	0.91429	0.8073	0.76923	1.0000	1.00000	0.87706
10	0.8529	0.68401	0.86364	0.83333	0.90476	0.88396	0.88235	0.7323	0.95455	0.7500	0.87500	0.91391
mean	0.8951	0.78410	0.87471	0.92495	0.93933	0.90477	0.87269	0.7335	0.87232	0.8663	0.90276	0.88464
median	0.9062	0.81034	0.87299	0.93213	0.93750	0.90330	0.87868	0.7438	0.87302	0.8990	0.93934	0.88707
std	0.0442	0.08832	0.07245	0.07624	0.05949	0.03394	0.05534	0.1125	0.08635	0.1079	0.08982	0.05039
	**RBS**						**SVM**					
**Fold**	**Acc**	**Kp**	**Se**	**Sp**	**Pr**	**G**	**Acc**	**Kp**	**Se**	**Sp**	**Pr**	**G**
1	0.9091	0.8121	0.85714	0.9474	0.92308	0.88950	0.96970	0.93738	0.92857	1.0000	1.00000	0.96362
2	0.9444	0.8831	0.95455	0.9286	0.95455	0.95455	0.88889	0.77215	0.86364	0.9286	0.95000	0.90579
3	0.9474	0.8939	0.88889	1.0000	1.00000	0.94281	0.97368	0.94708	0.94444	1.0000	1.00000	0.97183
4	0.9032	0.8058	0.93750	0.8667	0.88235	0.90951	0.90323	0.80585	0.93750	0.8667	0.88235	0.90951
5	0.8750	0.7277	0.82609	1.0000	1.00000	0.90889	0.96875	0.92523	0.95652	1.0000	1.00000	0.97802
6	0.9412	0.8824	0.94118	0.9412	0.94118	0.94118	0.94118	0.88235	0.94118	0.9412	0.94118	0.94118
7	0.8710	0.7293	0.85000	0.9091	0.94444	0.89598	0.87097	0.73950	0.80000	1.0000	1.00000	0.89443
8	0.8125	0.6206	0.78947	0.8462	0.88235	0.83462	0.93750	0.87352	0.89474	1.0000	1.00000	0.94591
9	0.9143	0.8135	0.84615	0.9545	0.91667	0.88070	0.91429	0.80734	0.76923	1.0000	1.00000	0.87706
10	0.8824	0.7323	0.95455	0.7500	0.87500	0.91391	0.85294	0.68401	0.86364	0.8333	0.90476	0.88396
mean	0.9000	0.7901	0.88455	0.9144	0.93196	0.90716	0.92211	0.83744	0.88995	0.9570	0.96783	0.92713
median	0.9062	0.8090	0.87302	0.9349	0.93213	0.90920	0.92589	0.84043	0.91165	1.0000	1.00000	0.92534
std	0.0417	0.0875	0.05935	0.0762	0.04532	0.03516	0.04297	0.08965	0.06485	0.0629	0.04536	0.03753

**Table 8:** Results obtained from the 5 × 2 cv when the threshold is set to 2.5× *g*: the different statistics are the Accuracy (Acc), Kappa factor (Kp), Sensitivity (Se), Specificity (Sp), Precision (Pr) and the geometric mean (G), all of them computed using Equations (1) to (8). The models are feed forward NN, Support Vector Machine (SVM), decision trees learned with C5.0 (DT) and Rule-Based systems learned with C5.0 (RBS).

	**NN**						**DT**					
**Fold**	**Acc**	**Kp**	**Se**	**Sp**	**Pr**	**G**	**Acc**	**Kp**	**Se**	**Sp**	**Pr**	**G**
1	0.8966	0.7930	0.8989	0.8941	0.8989	0.8989	0.9310	0.8621	0.9101	0.9529	0.9529	0.9313
2	0.8395	0.6763	0.8105	0.8806	0.9059	0.8569	0.8580	0.7105	0.8526	0.8657	0.9000	0.8760
3	0.8757	0.7508	0.8587	0.8961	0.9081	0.8830	0.8876	0.7736	0.8913	0.8831	0.9011	0.8962
4	0.8503	0.7022	0.7935	0.9200	0.9241	0.8563	0.8862	0.7704	0.8913	0.8800	0.9011	0.8962
5	0.8970	0.7935	0.8602	0.9444	0.9524	0.9051	0.9091	0.8189	0.8495	0.9861	0.9875	0.9159
6	0.8421	0.6855	0.7912	0.9000	0.9000	0.8439	0.8421	0.6846	0.8132	0.8750	0.8810	0.8464
7	0.8084	0.6211	0.6667	0.9625	0.9508	0.7962	0.8383	0.6785	0.7586	0.9250	0.9167	0.8339
8	0.9290	0.8548	0.9381	0.9167	0.9381	0.9381	0.9112	0.8188	0.9175	0.9028	0.9271	0.9223
9	0.8862	0.7724	0.8652	0.9103	0.9167	0.8906	0.9162	0.8324	0.8876	0.9487	0.9518	0.9192
10	0.8935	0.7881	0.8316	0.9730	0.9753	0.9006	0.9053	0.8088	0.8947	0.9189	0.9341	0.9142
mean	0.8718	0.7438	0.8315	0.9198	0.9270	0.8770	0.8885	0.7759	0.8667	0.9138	0.9253	0.8952
median	0.8810	0.7616	0.8451	0.9135	0.9204	0.8868	0.8965	0.7912	0.8895	0.9109	0.9219	0.9052
std	0.0359	0.0706	0.0740	0.0308	0.0261	0.0398	0.0323	0.0646	0.0494	0.0397	0.0321	0.0332
	**RBS**						**SVM**					
**Fold**	**Acc**	**Kp**	**Se**	**Sp**	**Pr**	**G**	**Acc**	**Kp**	**Se**	**Sp**	**Pr**	**G**
1	0.9195	0.8392	0.8989	0.9412	0.9412	0.9198	0.9253	0.8509	0.8764	0.9765	0.9750	0.9244
2	0.8210	0.6381	0.8000	0.8508	0.8837	0.8408	0.88272	0.7619	0.8632	0.9105	0.9318	0.8967
3	0.8698	0.7381	0.8696	0.8701	0.8889	0.8792	0.9172	0.8341	0.8913	0.9481	0.9535	0.9219
4	0.8982	0.7945	0.9022	0.8933	0.9121	0.9071	0.8982	0.7970	0.8478	0.9600	0.9630	0.9036
5	0.8485	0.7000	0.7742	0.9444	0.9474	0.8564	0.9212	0.8416	0.8925	0.9583	0.9651	0.9281
6	0.8304	0.6622	0.7802	0.8875	0.8875	0.8321	0.8421	0.6836	0.8352	0.8500	0.8636	0.8493
7	0.8204	0.6430	0.7356	0.9125	0.9014	0.8143	0.8503	0.7026	0.7586	0.9500	0.9429	0.8458
8	0.9112	0.8214	0.8763	0.9583	0.9659	0.9200	0.9468	0.8909	0.9588	0.9306	0.9490	0.9539
9	0.9042	0.8082	0.8876	0.9231	0.9294	0.9083	0.8982	0.7963	0.8764	0.9231	0.9286	0.9021
10	0.8994	0.7984	0.8632	0.9460	0.9535	0.9072	0.8994	0.7996	0.8421	0.9730	0.9756	0.9064
mean	0.8723	0.7443	0.8388	0.9127	0.9211	0.8785	0.8981	0.7958	0.8642	0.9380	0.9448	0.9032
median	0.8840	0.7663	0.8664	0.9178	0.9208	0.8932	0.8988	0.7983	0.8698	0.9490	0.9512	0.9050
std	0.0392	0.0780	0.0603	0.0362	0.0303	0.0396	0.0328	0.0650	0.0511	0.0375	0.0328	0.0337

**Table 9:** Results obtained from the 5 × 2 cv when the threshold is set to 3.0× *g*: the different statistics are the Accuracy (Acc), Kappa factor (Kp), Sensitivity (Se), Specificity (Sp), Precision (Pr) and the geometric mean (G), all of them computed using Equations (1) to (8). The three different models are feed forward NN, Support Vector Machines (SVM), Decision Trees learned with C5.0 (DT) and Rule-Based Systems learned with C5.0 (RBS).

	**NN**						**DT**					
**Fold**	**Acc**	**Kp**	**Se**	**Sp**	**Pr**	**G**	**Acc**	**Kp**	**Se**	**Sp**	**Pr**	**G**
1	0.8781	0.7564	0.8352	0.9315	0.9383	0.8852	0.9268	0.8535	0.8901	0.9726	0.9759	0.9320
2	0.9302	0.8603	0.9032	0.9620	0.9655	0.9339	0.9070	0.8131	0.9032	0.9114	0.9231	0.9131
3	0.9203	0.8387	0.9032	0.9429	0.9546	0.9285	0.9141	0.8278	0.8710	0.9714	0.9759	0.9219
4	0.9075	0.8156	0.8571	0.9634	0.9630	0.9085	0.9249	0.8490	0.9451	0.9024	0.9149	0.9299
5	0.9042	0.8064	0.9130	0.8933	0.9130	0.9130	0.8922	0.7832	0.8804	0.9067	0.9205	0.9002
6	0.9053	0.8100	0.8913	0.9221	0.9318	0.9113	0.9053	0.8108	0.8696	0.9481	0.9524	0.9100
7	0.8795	0.7568	0.8901	0.8667	0.8901	0.8901	0.8675	0.7362	0.8132	0.9333	0.9367	0.8728
8	0.9000	0.8002	0.8602	0.9481	0.9524	0.9051	0.8941	0.7882	0.8602	0.9351	0.9412	0.8998
9	0.9102	0.8178	0.9348	0.8800	0.9053	0.9199	0.9042	0.8078	0.8804	0.9333	0.9419	0.9106
10	0.9349	0.8695	0.9130	0.9610	0.9655	0.9389	0.8935	0.7875	0.8478	0.9481	0.9512	0.8980
mean	0.9070	0.8132	0.8901	0.9271	0.9379	0.9135	0.90230	0.8057	0.8761	0.9362	0.9434	0.9088
median	0.9064	0.8128	0.8973	0.9372	0.9453	0.9122	0.9048	0.8093	0.8757	0.9342	0.9415	0.9103
std	0.0187	0.0376	0.0305	0.0356	0.0272	0.0176	0.0175	0.0345	0.0347	0.0247	0.0212	0.0174
	**RBS**						**SVM**					
**Fold**	**Acc**	**Kp**	**Se**	**Sp**	**Pr**	**G**	**Acc**	**Kp**	**Se**	**Sp**	**Pr**	**G**
1	0.9024	0.8041	0.8791	0.9315	0.9412	0.9096	0.9451	0.8888	0.9560	0.9315	0.9457	0.9508
2	0.9128	0.8246	0.9140	0.9114	0.9239	0.9189	0.8954	0.7905	0.8710	0.9241	0.9310	0.9005
3	0.9203	0.8398	0.8817	0.9714	0.9762	0.9278	0.9080	0.8125	0.9140	0.9000	0.9239	0.9189
4	0.9364	0.8724	0.9451	0.9268	0.9348	0.9399	0.9017	0.8035	0.8791	0.9268	0.9302	0.9043
5	0.8802	0.7586	0.8804	0.8800	0.9000	0.8902	0.9222	0.8421	0.9457	0.8933	0.9158	0.9306
6	0.8994	0.7987	0.8696	0.9351	0.9412	0.9047	0.9112	0.8205	0.9348	0.8831	0.9053	0.9199
7	0.8735	0.7473	0.8352	0.9200	0.9268	0.8798	0.9217	0.8406	0.9670	0.8667	0.8980	0.9319
8	0.9000	0.7989	0.8925	0.9091	0.9222	0.9072	0.8941	0.7887	0.8495	0.9481	0.9518	0.8992
9	0.8922	0.7838	0.8696	0.9200	0.9302	0.8994	0.9281	0.8544	0.9457	0.9067	0.9255	0.9355
10	0.8994	0.7987	0.8696	0.9351	0.9412	0.9047	0.9349	0.8692	0.9239	0.9481	0.9551	0.9394
mean	0.9017	0.8027	0.8837	0.9240	0.9338	0.9082	0.9162	0.8311	0.9187	0.9128	0.9282	0.9231
median	0.8997	0.7988	0.8798	0.9234	0.9325	0.9059	0.9165	0.8306	0.9294	0.9154	0.9279	0.9253
std	0.0184	0.0367	0.0293	0.0234	0.0194	0.0175	0.0171	0.0337	0.0396	0.0274	0.0189	0.0176

**Table 10:** Results obtained from the 5 × 2 cv when the threshold is set to 3.09290× *g*: the different statistics are the Accuracy (Acc), Kappa factor (Kp), Sensitivity (Se), Specificity (Sp), Precision (Pr) and the Geometric mean (G), all of them computed using Equations (1) to (8). The three different models are feed forward NN, Support Vector Machines (SVM), Decision Trees learned with C5.0 (DT) and Rule-Based Systems learned with C5.0 (RBS).

	**NN**						**DT**					
**Fold**	**Acc**	**Kp**	**Se**	**Sp**	**Pr**	**G**	**Acc**	**Kp**	**Se**	**Sp**	**Pr**	**G**
1	0.9226	0.8440	0.9239	0.9211	0.9341	0.9290	0.8988	0.7936	0.9565	0.8290	0.8713	0.9129
2	0.8810	0.7586	0.9130	0.8421	0.8750	0.8938	0.9286	0.8548	0.9674	0.8816	0.9082	0.9373
3	0.8810	0.7608	0.8696	0.8947	0.9091	0.8891	0.8333	0.6549	0.9674	0.6711	0.7807	0.8691
4	0.8750	0.7468	0.9022	0.8421	0.8737	0.8878	0.9048	0.8065	0.9457	0.8553	0.8878	0.9163
5	0.8988	0.7969	0.8804	0.9211	0.9310	0.9054	0.7798	0.5540	0.8152	0.7368	0.7895	0.8022
6	0.8869	0.7725	0.8804	0.8947	0.9101	0.8952	0.8810	0.7603	0.8804	0.8816	0.9000	0.8902
7	0.8988	0.7965	0.8913	0.9079	0.9214	0.9062	0.8691	0.7405	0.7935	0.9605	0.9605	0.8730
8	0.8810	0.7624	0.8370	0.9342	0.9390	0.8865	0.9226	0.8426	0.9674	0.8684	0.8990	0.9326
9	0.8691	0.7351	0.8913	0.8421	0.8723	0.8818	0.8929	0.7838	0.9022	0.8816	0.9022	0.9022
10	0.8750	0.7509	0.8261	0.9342	0.9383	0.8804	0.9167	0.8323	0.9130	0.9211	0.9333	0.9231
mean	0.8869	0.7725	0.8815	0.8934	0.9104	0.8955	0.8827	0.7623	0.9109	0.8487	0.8832	0.8959
median	0.8810	0.7616	0.8859	0.9013	0.9157	0.8915	0.8958	0.7887	0.9294	0.8750	0.8995	0.9075
std	0.0159	0.0320	0.0309	0.0380	0.0274	0.0147	0.0459	0.0935	0.0640	0.0856	0.0572	0.0403
	**RBS**						**SVM**					
**Fold**	**Acc**	**Kp**	**Se**	**Sp**	**Pr**	**G**	**Acc**	**Kp**	**Se**	**Sp**	**Pr**	**G**
1	0.9107	0.8200	0.9130	0.9079	0.9231	0.9181	0.9345	0.8671	0.9674	0.8947	0.9175	0.9421
2	0.8929	0.7847	0.8804	0.9079	0.9205	0.9002	0.8988	0.7955	0.9130	0.8816	0.9032	0.9081
3	0.8869	0.7694	0.9457	0.8158	0.8614	0.9025	0.9107	0.8200	0.9130	0.9079	0.9231	0.9181
4	0.8988	0.7936	0.9565	0.8290	0.8713	0.9129	0.9048	0.8069	0.9348	0.8684	0.8958	0.9151
5	0.8691	0.7387	0.8261	0.9211	0.9268	0.8750	0.9107	0.8192	0.9348	0.8816	0.9053	0.9199
6	0.9107	0.8196	0.9239	0.8947	0.9141	0.9189	0.8869	0.7730	0.8696	0.9079	0.9195	0.8942
7	0.8631	0.7290	0.7826	0.9605	0.9600	0.8668	0.9405	0.8790	0.9787	0.8947	0.9184	0.9478
8	0.9286	0.8545	0.9783	0.8684	0.9000	0.9383	0.8988	0.7951	0.9239	0.8684	0.8947	0.9092
9	0.9107	0.8196	0.9239	0.8947	0.9140	0.9189	0.9107	0.8183	0.9565	0.8553	0.8889	0.9221
10	0.9286	0.8562	0.9239	0.9342	0.9444	0.9341	0.9167	0.8326	0.9022	0.9342	0.9432	0.9225
mean	0.9000	0.7985	0.9054	0.8934	0.9135	0.9086	0.9113	0.8207	0.9294	0.8895	0.9110	0.9199
median	0.9048	0.8066	0.9239	0.9013	0.9172	0.9155	0.9107	0.8188	0.9294	0.8882	0.9114	0.9190
std	0.0224	0.0438	0.0602	0.0449	0.0300	0.0232	0.0162	0.0324	0.0325	0.0234	0.0164	0.0157

**Table 11:** Confusion matrices for the three analyzed thresholds and for each model type: feed forward NN, Decision Trees learned with C5.0 (DT), Rule-Based Systems learned with C5.0 (RBS) and Support Vector Machines (SVM).

**Threshold 2.5**											
	**Reference**			**Reference**			**Reference**			**Reference**	
**NN**	**Fall**	**Not Fall**	**DT**	**Fall**	**Not Fall**	**RBS**	**Fall**	**Not Fall**	**SVM**	**Fall**	**Not Fall**
**Fall**	10	47	**Fall**	10	20	**Fall**	10	42	**Fall**	8	18
**Not Fall**	2	250	**Not Fall**	2	277	**Not Fall**	2	245	**Not Fall**	4	279
**Threshold 3.0**											
	**Reference**			**Reference**			**Reference**			**Reference**	
**NN**	**Fall**	**Not Fall**	**DT**	**Fall**	**Not Fall**	**RBS**	**Fall**	**Not Fall**	**SVM**	**Fall**	**Not Fall**
**Fall**	12	52	**Fall**	11	18	**Fall**	11	29	**Fall**	10	12
**Not Fall**	0	245	**Not Fall**	1	279	**Not Fall**	1	268	**Not Fall**	2	285
**Threshold 3.09290**											
	**Reference**			**Reference**			**Reference**			**Reference**	
**NN**	**Fall**	**Not Fall**	**DT**	**Fall**	**Not Fall**	**RBS**	**Fall**	**Not Fall**	**SVM**	**Fall**	**Not Fall**
**Fall**	12	59	**Fall**	11	26	**Fall**	12	35	**Fall**	10	13
**Not Fall**	0	238	**Not Fall**	1	271	**Not Fall**	0	262	**Not Fall**	2	284

**Table 12:** Results obtained for the best model for each threshold. Different statistics are shown: the Accuracy (Acc), Kappa factor (Kp), Sensitivity (Se), Specificity (Sp), Precision (Pr) and the Geometric mean (G), all of them computed using Equations (1) to (8). The models are feed forward NN, Decision Trees learned with C5.0 (DT), Rule-Bases Systems learned with C5.0 (RBS) and Support Vector Machines (SVM).

Threshold	Model	Acc	Kp	Se	Sp	Pr	G
2.5	NN	0.8414	0.2412	0.8333	0.8418	0.1754	0.8375
	DT	0.9288	0.4454	0.8333	0.9327	0.3333	0.8816
	RBS	0.8576	0.2662	0.8333	0.8586	0.1923	0.8459
	SVM	0.9288	0.3886	0.6667	0.9394	0.3077	0.7914
3.0	NN	0.8317	0.2679	1.0000	0.8249	0.1875	0.9082
	DT	0.9385	0.5096	0.9167	0.9394	0.3793	0.9280
	RBS	0.9029	0.3864	0.9167	0.9024	0.2750	0.9095
	SVM	0.9547	0.5664	0.8333	0.9596	0.4545	0.8942
3.09290	NN	0.8091	0.2386	1.0000	0.8013	0.1690	0.8952
	DT	0.9126	0.4146	0.9167	0.9125	0.2973	0.9146
	RBS	0.8867	0.3677	1.0000	0.8822	0.2553	0.9392
	SVM	0.9515	0.5484	0.8333	0.9562	0.4348	0.8927

## Abbreviations

The following abbreviations are used in this manuscript:

3DACC	Tri-Axial Accelerometer
Acc	Accuracy
ADL	Activity of Daily Living
AI	Artificial Intelligence
BLE	Bluetooth Low Energy
cv	Cross Validation
DT	Decision Tree
FD	Fall Detection
FN	False Negative
FP	False Positive
K	Cohen’s Kappa measurement
ML	Machine Learning
NN	Neural network
RBS	Rule-Based System
Sen	Sensitivity
Spec	Specificity
SVM	Support Vector Machine
TS	Time Series
TP	True Positive
TN	True Negative
Std	Standard Deviation
AAMV	Absolute Acceleration Magnitude Module
ARI	Activity Radio Index
FFI	Free Fall Index
IDI	Impact Duration Index
MPI	Maximum Peak Index
MVI	Minimum Valley Index
PDI	Peak Duration Index
SCI	Step Count Index
